# Supplementary material for: Waste animal fat with hydrothermal liquefaction as a potential route to marine biofuels
Source: PeerJ. 2023 Dec 18;11:e16504. doi: 10.7717/peerj.16504 (PMC10734409; doi:10.7717/peerj.16504)
Supplement: Supplemental Information 1 [file peerj-11-16504-s001.docx]

**Supplementary Information**

**Waste animal fat with hydrothermal liquefaction**

**as a potential route to marine biofuels**

Efraim Steinbruch^1^^, Siddaq Singh^1^^, Maya Mosseri^1^, Michael Epstein ^1^, Abraham Kribus^2^, Michael Gozin^3,4,5^, Dušan Drabik^6^, Alexander Golberg^1^*

**Figure S1**. Schematic diagram of the HTL reactor.

**
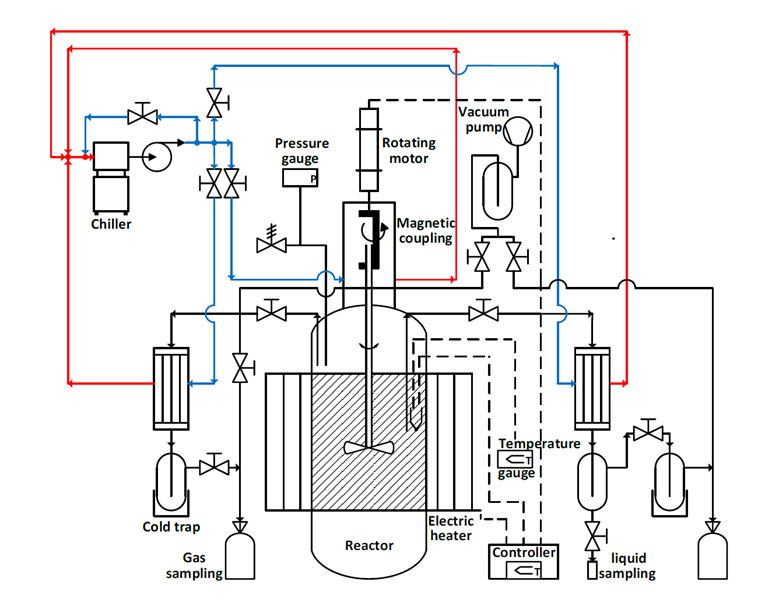
**

**Figure S2.**  (a) DTG and (b) TGA curves of untreated feedstock and its bio-oils obtained at 330^o^C under different solid loads and retention times.

**(a)**

**(b)**


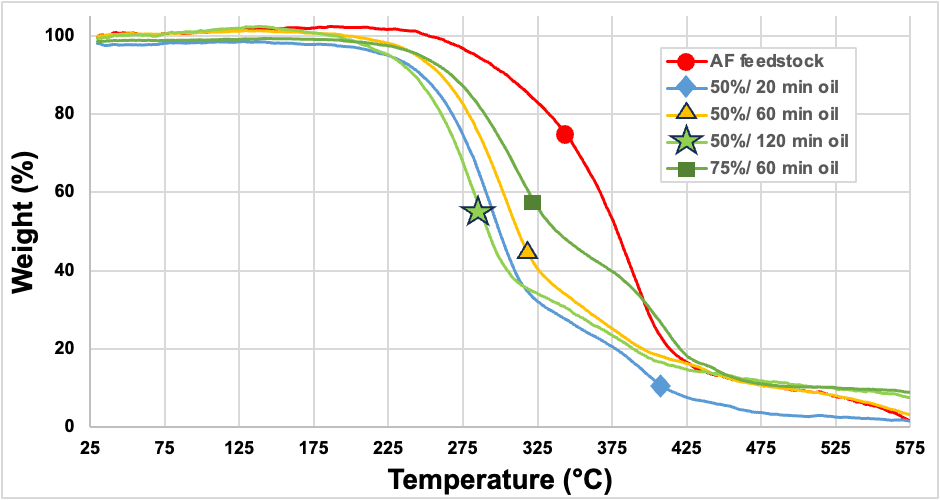


**Figure S3**. FTIR spectra of untreated feedstock and its HTL bio-oils obtained at 330^o^C under different solid loads and retention times. A clear difference between the feedstock (red) and the bio-crudes (other colors is seen).

**
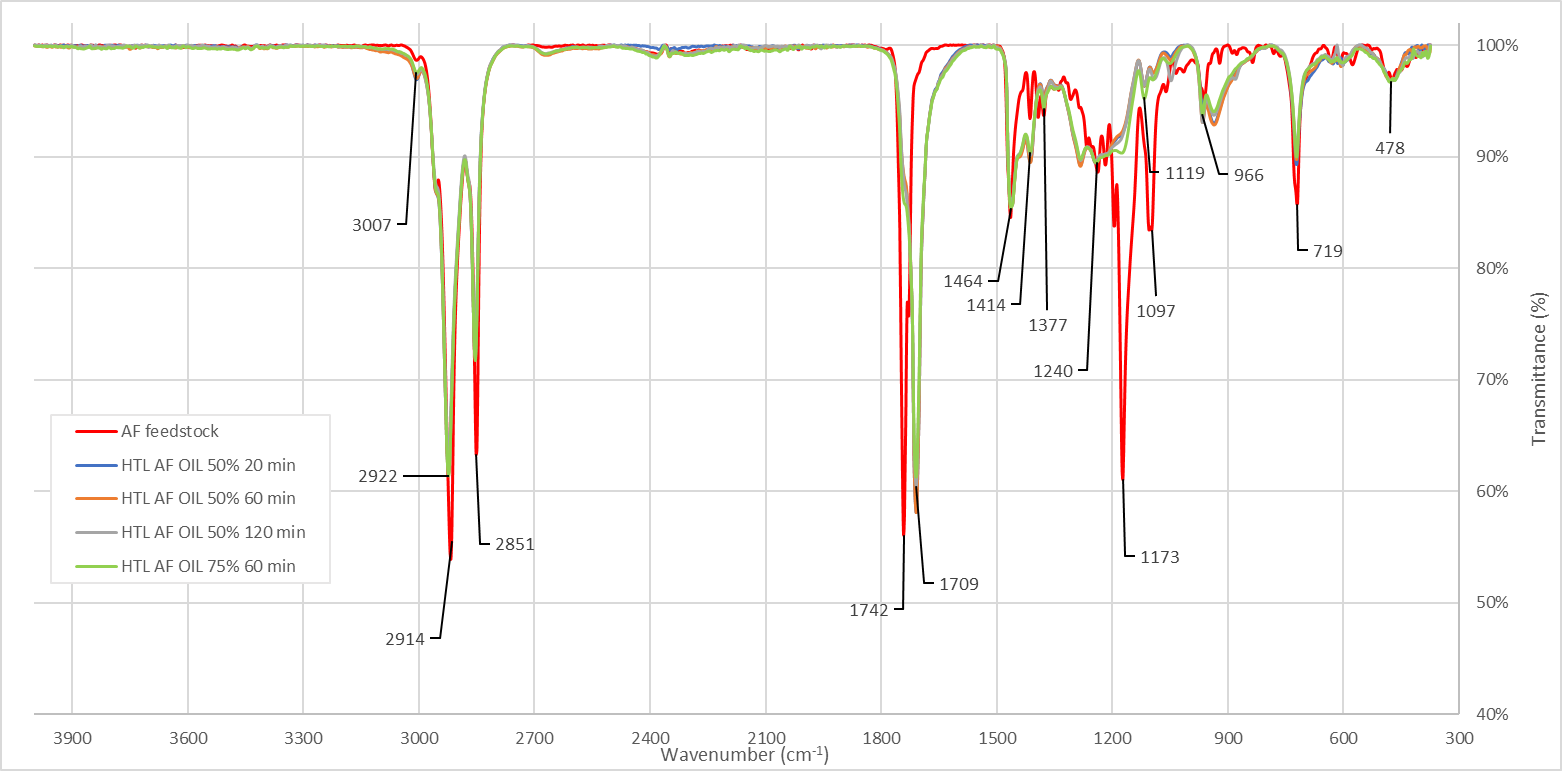
**

**Table S1.** Elemental analysis, high heating values (HHV), and density of feedstock and bio-oil samples.

| **Sample Name** | **Temp. (**ºC**) / RT (min)** | **Solid Load (%)** | **Density of Bio-oil**  **(g/cm^3^)** | **N** | **C** | **H** | **S** | **O** | **HHV (MJ/kg)** |
| --- | --- | --- | --- | --- | --- | --- | --- | --- | --- |
| Animal Fat feed-stock | - | - |  | 0 | 75.8± 1.3 | 12.0± 0.7 | 0.016 | 12.3± 0.9 | 39.6 ± 0.2 |
| Bio-oil | 330/20 | 50 | 0.9± 0.1 | 0 | 74.2± 2.1 | 11.9± 0.6 | 0.016 | 14.0± 1.2 | 38.7 ± 0.4 |
| Solid residue | 330/20 | 50 | - | - | - | - |  | - | 38 ± 1 |

**Table S2**. Trace elements analysis in animal fat-derived bio-oil by HTL 330 °C, 60 min retention time and 50% solid load.

| **Element** | **Concentration mg/kg** | **Element** | **Concentration mg/kg** |
| --- | --- | --- | --- |
| Ag Silver | <1 | Mo Molybdenum | <1 |
| Al Aluminium | <1 | Na Sodium | 2 |
| K Potassium | <1 | Ni Nickel | 5 |
| Ba Barium | <1 | P Phosphorus | 1 |
| Ca Calcium | 10 | Pb Lead | <1 |
| Cd Cadmium | <1 | Si Silicon | 3 |
| Cr Chromium | 1 | Sn Tin | <1 |
| Cu Copper | 2 | Ti Titanium | <1 |
| Fe Iron | 82 | V Vanadium | <1 |
| Mg Magnesium | 1 | Zn Zinc | 2 |
| Mn Manganese | <1 | S Sulphur | 160 |

**Table S3. Input-output parameters for the economic model.** The parameters are related to a lab-scale experimental setup that uses 0.5 kg of animal fat waste to produce 0.133 kg bio-oil as the main product by HTL 330 °C, 60 min, 50% solid load, and 0.337 kg of solid residue. The process is energetical sustainable.

| **Stage** | **In/Out** | **Utility/Material** | **Unit** | **Base-case** |
| --- | --- | --- | --- | --- |
| Feedstock | input | animal fat waste | kg | 0.500 |
|  |  | water | kg | 0.500 |
| HTL process  Temperature/retention time (330 °C/ 60min) | input* | Heat required for the reaction supplied by a portion of the solid residue | MJ | 1.089 |
|  | input* | A portion of the solid fat residue from the process (based on 36 MJ/kg LHV) | kg | 0.030 |
| Electricity (LHV=36 MJ/kg)** | input | Heat from combusting solid fat residue | MJ | 12.123 |
|  | input | A portion of the solid fat residue from the process available for electricity | kg | 0.307 |
|  | output*** | Electricity | MJ  kWh | 3.637  1.01 |
| Bio-oil production | output | bio-oil | kg | 0.133 |
|  | output | water | kg | 0.560 |
|  | output | Total solid fat residue | kg | 0.337 |

*Needed heat for reactor 25 C to 330 C: Heating 0.5 kg of water (enthalpy difference of saturated liquid): 0.638 MJ. Heating of 0.5 kg AF (Cp = 2.96 kJ/kgK^-1^): 0.451 MJ. Total heat needed: 1.09 MJ. The needed amount of solid combustion according to 36 MJ/kg: 0.03 kg.

** Dulong’s formula, calculated from HHV.

*** Assuming 30% energy conversion from heat to electricity.

**References**

1. Thiab, R., Amin, M. & Umar, H. Thermal Properties of Beef Tallow/Coconut Oil Bio PCM Using T-History Method for Wall Building Applications. *European Journal of Engineering Research and Science* **4**, 38–40 (2019).
